# NIA DIVISION OF EXTRAMURAL ACTIVITIES

**DOI:** 10.1093/geroni/igae098.0335

**Published:** 2024-12-31

**Authors:** Kenneth Santora

**Affiliations:** National Institute on Aging, Baltimore, Maryland, United States

## Abstract

Dr. Santora will discuss the work of the NIA Division of Extramural Activities. Dr. Santora will also be available for small group discussion.

